# Comment to: Qi, Yi, et al. “Bibliometric Analysis of Algal-Bacterial Symbiosis in Wastewater Treatment”, *Int. J. Environ. Res. Public Health* 2019, *16*, 1077

**DOI:** 10.3390/ijerph16112034

**Published:** 2019-06-07

**Authors:** Yuh-Shan Ho

**Affiliations:** Trend Research Centre, Asia University, No. 500, Lioufeng Road, Wufeng, Taichung County 41354, Taiwan; ysho@asia.edu.tw; Tel.: +886-4-2332-3456 (ext. 1797); Fax: +886-4-2330-583

Qi et al. recently published an article in the journal, entitled “Bibliometric Analysis of Algal–Bacterial Symbiosis in Wastewater Treatment” [1]. The results presented in the original paper are not acceptable because of the use of inappropriate methods. The authors mentioned in 2.1. Data Sources that “To obtain reliable and accurate details on the topic of algal-bacterial symbiosis in wastewater treatment, 940 publications were obtained on 16 September 2018, using (alga* or micro alga* or micro-alga* or microalga* or *alga*-bacteri* consorti*) and (bacteri* or activated sludge) and (wastewater or sewage) as the search query from Science Citation Index Expanded (SCI-EXPANDED) database for the period from 1998 to 2017.” There are searching keywords problems as follows:

1. (alga* or micro alga* or micro-alga*) is the same as (alga*)
2. (*alga*-bacteri* consorti*) is the same as (*alga*-bacteri* and consorti*)
3. (activated sludge) is the same as (activated and sludge)
4. Results by using searching keywords (alga* or microalga* or (*alga*-bacteri* and consorti*)) and (bacteri* or (activated and sludge)) and (wastewater or sewage) are the same as those from the original paper [1].

Using the same method from the original paper [1], 937 documents were found. SCI-EXPANDED is designed for researchers to find published literature but not used for bibliometric studies [2,3,4]. Thus, it is always necessary to use an accurate bibliometric method when using SCI-EXPANDED [2,3,4]. It was pointed out that the documents, which can only be searched out by *KeyWords Plus*, were irrelevant to “algal-bacterial symbiosis in wastewater treatment” [5]. Ho’s group was the first to propose “front page” as a filter to improve the bibliometric method [6,7,8]. Only documents with searching keywords in their “front page”, including the article title, the abstract, and the author keywords were considered. As a result, 681 documents (73% of 937 documents) had searching keywords in their “front page”, while 256 documents (27%) did not include searching keywords in their “front page”—for example, a highly cited review with 100 or more total citations from Web of Science Core Collection since its publication and to the end of 2017 (*TC*_2017_ ≥ 100) [9], “Recent advances in removing phosphorus from wastewater and its future use as fertilizer (1997–2003)” [10], and highly cited article “Treatment of micropollutants in municipal wastewater: Ozone or powdered activated carbon?” [11]. These results show the huge difference from the results in the original paper [1]. By using the “front page” as a filter, introducing unrelated articles for analysis can be avoided [6,12]. In recent years, similar rebuttals have also been published in *Environmental Science and Pollution Research* [2], *Renewable and Sustainable Energy Reviews* [12], and *Journal of Soils and Sediments* [4].

Research is the way to the truth, so innovations are important to find something new or a new understanding to approach the truth [4]. It is necessary to improve a researcher’s use of methods and concepts in order to have accurate results and discussions [4]. However, Qi et al. used inappropriate methods to publish a bibliometric paper in *Int. J. Environ. Res. Public Health*, which may result in misleading the journal readers [3,4]. From my point of view, Qi et al. should have understood SCI-EXPANDED and thus provided a greater accuracy and information about their data. Furthermore, using such a limited number of publications for bibliometric studies is inappropriate from a statistical point of view.

## References

[1] Qi Y., Chen X.Y., Hu Z., Song C.F., Cui Y.L. (2019). Bibliometric analysis of algal-bacterial symbiosis in wastewater treatment. Int. J. Environ. Res. Public Health.

[2] Ho Y.S. (2018). Comments on “Mapping the scientific research on non-point source pollution: A bibliometric analysis” by Yang et al. Environ. Sci. Pollut. Res..

[3] Ho Y.S. (2018). Comment on: “A Bibliometric Analysis and Visualization of Medical Big Data Research”. Sustainability.

[4] Ho Y.S. (2019). Some comments on: Mao et al. (2018) “Bibliometric analysis of insights into soil remediation”. J. Soils Sediments.

[5] Fu H.Z., Ho Y.S. (2015). Top cited articles in thermodynamic research. J. Eng. Thermophys..

[6] Fu H.Z., Wang M.H., Ho Y.S. (2012). The most frequently cited adsorption research articles in the Science Citation Index (Expanded). J. Colloid Interface Sci..

[7] Fu H.Z., Ho Y.S. (2014). Top cited articles in adsorption research using Y-index. Res. Evaluat..

[8] Ho Y.S., Fu H.Z. (2016). Mapping of metal-organic frameworks publications: A bibliometric analysis. Inorg. Chem. Commun..

[9] Hsu Y.H.E., Ho Y.S. (2014). Highly cited articles in health care sciences and services field in Science Citation Index Expanded: A bibliometric analysis for 1958-2012. Methods Inf. Med..

[10] de-Bashan L.E., Bashan Y. (2004). Recent advances in removing phosphorus from wastewater and its future use as fertilizer (1997–2003). Water Res..

[11] Margot J., Kienle C., Magnet A., Weil M., Rossi L., de Alencastro L.F., Abegglen C., Thonney D., Chèvre N., Schärer M. (2013). Treatment of micropollutants in municipal wastewater: Ozone or powdered activated carbon?. Sci. Total Environ..

[12] Ho Y.S. (2018). Comments on “Past, current and future of biomass energy research: A bibliometric analysis” by Mao et al. (2015). Renew. Sust. Energ. Rev..

